# Adult mesenchymal stem cells and their possibilities for Dentistry: what to expect?

**DOI:** 10.1590/2177-6709.25.3.085-092.sar

**Published:** 2020

**Authors:** José Ricardo Muniz Ferreira, Anna Paula Greck

**Affiliations:** 1 Instituto Militar de Engenharia (Rio de Janeiro/RJ, Brazil).; 2 University of Paris (Paris, France).

**Keywords:** Mesenchymal stem cells, Regenerative dentistry, Cell therapy, Tissue bioengineering, Cryopreservation

## Abstract

**Introduction:**

Stem cells obtained from the pulp of human deciduous teeth are highly proliferative and plastic multipotent cells, which makes them a relevant model of stem cells, applied in several biomedical areas, with different purposes.

**Objective:**

Based on a brief review of the literature, the present work intends to present from conceptual aspects about stem cells, classifications, potential (*in vitro* and *in vivo*) applications in dental practice, cell culture, cryopreservation and its importance, ethical and regulatory aspects, as well as the role of the dental surgeon as the endorser responsible for the entire clinical stage that involves the process of collecting stem cells obtained from dental pulps for cryopreservation, with a view to using them under appropriate conditions, in accordance with scientifically proven and justified good laboratory and clinical practices.

## RESUMO

**Introdução:**

As células-tronco obtidas a partir da polpa de dentes decíduos humanos são células multipotentes altamente proliferativas e plásticas, o que as torna um modelo relevante de células-tronco, aplicado em diversas áreas biomédicas, com diferentes propósitos.

**Objetivo:**

A partir de uma breve revisão da literatura, o presente trabalho pretende apresentar desde aspectos conceituais acerca das células-tronco, classificações, potenciais aplicações (*in vitro* e *in vivo*) na prática odontológica, cultivo celular, criopreservação e sua importância, aspectos éticos e regulatórios, bem como o papel do cirurgião-dentista como homologador responsável por toda a etapa clínica que envolve o processo de coleta das células-tronco obtidas a partir de polpas dentais para criopreservação, com vistas ao uso em condições adequadas, em acordo com as boas práticas laboratoriais e clínicas cientificamente comprovadas e justificadas.

## INTRODUCTION

The search for longevity associated with good quality of life has accompanied the history of humanity since the beginning of time. Over the past 50 years, we have increased our life expectancy, worldwide, by approximately 30 years.^1^ Much of this is due to small changes in habits and major scientific and clinical advances that have enabled the implementation of increasingly effective prevention, control and treatment measures for so-called infectious diseases. In those places where such measures have been successfully established, considering the socioeconomic and cultural aspects involved, these diseases today account for about 5% of the indices mortality.^2^ However, there is still much to be done, and today so-called degenerative diseases represent a major challenge to be overcome so that we can move forward with the purpose of living longer and living well.

There is no doubt about the important role played by Dentistry along this journey. After all, many of the pathologies that affect the stomatognathic system have decisive systemic consequences for the longevity-quality of life binomial to be enforced.

**Figure 1:**
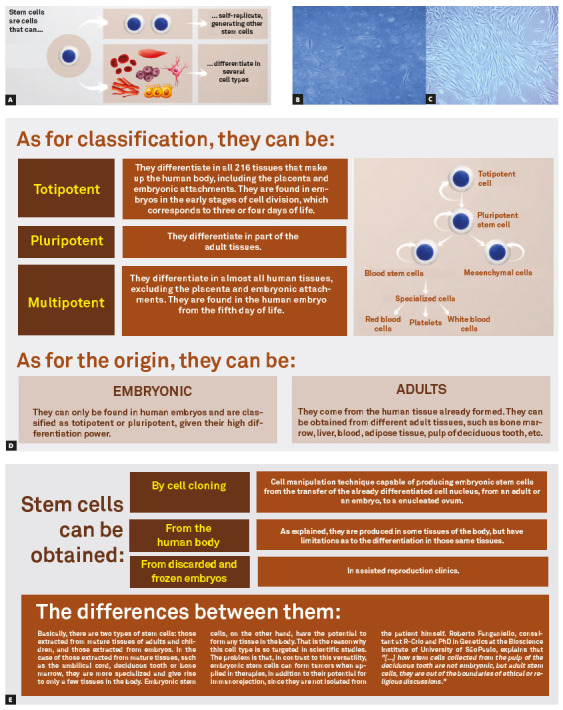
A) Stem cells are called nonspecific, and therefore have a great capacity for multiplication and differentiation. B) Image obtained by optical microscopy (10X), in which the presence of mesenchymal stem cells isolated from a deciduous dental pulp (4 days of culture) can be observed. C) Image obtained by optical microscopy (10X), in which the presence of isolated and expanded mesenchymal stem cells can be observed in the laboratory (12 days of culture, in confluence). D) Classification of stem cells according to their potential for differentiation and origin. E) Ways to obtain stem cells and differences between adult and embryonic stem cells.

From the twentieth century, Cell Biology and Genetics began to contribute as diagnostic tools and therapies aimed at treating many of the ills that afflict humanity. In the 50s, it was carried out, by Edward Donnall Thomas, the first bone marrow transplant. In 1961, James Till and Ernest McCulloch pointed to the existence of hematopoietic stem cells in the bone marrow of mice, opening a perspective for understanding the mechanisms involved in these transplants.^3^ Between the 70s and 80s, Professor Alexander Friedenstein's team, studying mesenchymal stem cells, demonstrated the capacity for self-renewal and differentiation of these cells, attracting attention from the scientific community to the need to promote research aimed at the use, in a targeted and predictable way, of this biological material, in relation to embryonic stem cells already known previously (Fig 1). 

Adult mesenchymal stem cells present themselves as an important instrument within the fields of Regenerative Medicine and Dentistry, as they are cells that, despite being nonspecific, have a great capacity for multiplication, differentiation into specialized cells and constituents of specialized tissues and with immunomodulatory competence. There are reports of a large number of places from which adult mesenchymal stem cells can be isolated, such as: bone marrow,^4^ adipose tissue,^5^ musculoskeletal tissue^6^, labial orbicularis muscle^7^, dental pulp^8^, dermis^9^ and pulp of primary teeth.^10^

## STEM CELLS AND THEIR POSSIBILITIES IN REGENERATIVE DENTISTRY AND MEDICINE

**Figure 2:**
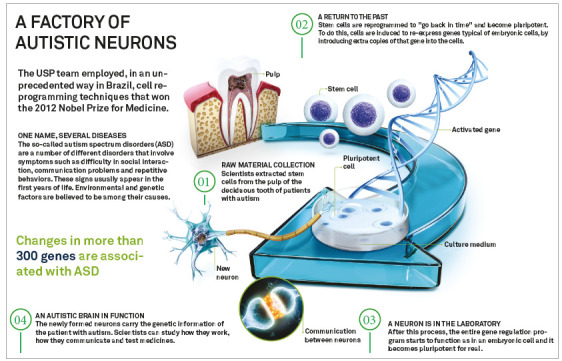
Infogram based on the article by Griesi-Oliveira et al.^12^

**Figure 3:**
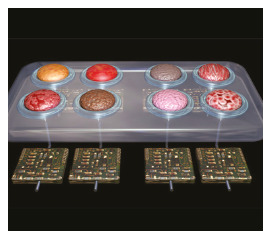
Microfluidics: technology that allows creating organs on chips. Source: Bombaldi,^13^ 2019.

**Figure 4:**
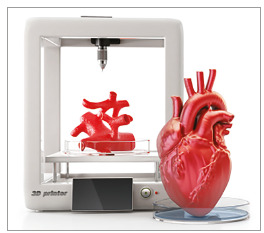
Generation of organs in the laboratory.

**Figure 5:**
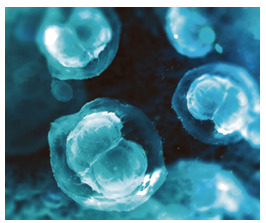
Stem cells in cell therapy.

1. Stratification of patients: use of stem cells to recreate a specific disease in the laboratory (Alzheimer's, Autism, Parkinson, etc.) and, thus, allow a more detailed investigation of the disease, so that new treatments can be developed (Fig 2).
2. Drug development and testing: use of stem cells to generate tissues or organs of interest for the development and testing of new drugs, allowing for a local and systemic evaluation without the need for tests on animals and humans (Fig 3).
3. Generation of functional organs in the laboratory: by directing the differentiation of stem cells associated with a specific framework (previously decellularized organ), generating functional and immunologically compatible organs^14^ ^-^ ^16^ (Fig 4).
4. Cell therapy: by transplanting or injecting live stem cells, differentiated or not, into a patient, in order to replace or regenerate damaged cells or tissues (Fig 5).

## MESENCHYMAL STEM CELLS OF DECIDUOUS PULPS: WHAT THE DENTAL SURGEON NEEDS TO KNOW

**Figure 6:**
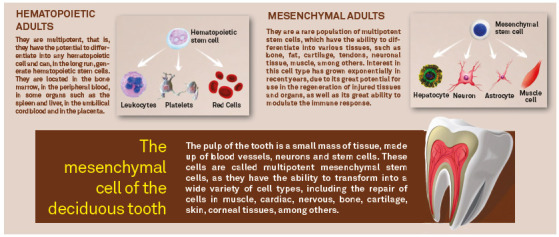
Comparative illustration between adult hematopoietic and mesenchymal stem cells.

Adult stem cells are classified, according to their origin, into hematopoietic and mesenchymal (Fig 6). Stem cells of hematopoietic origin are able to differentiate into specialized cells of white and red blood lines, while mesenchymal cells are able to differentiate into specialized cells that form hard tissues and organs, such as bones, cartilages, muscles, nerves, among others (Fig 6).

**Figure 7:**
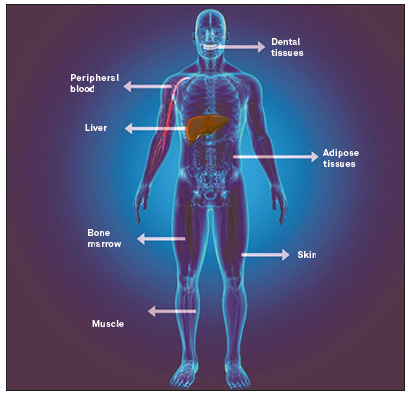
Sources of mesenchymal stem cells.

The stem cells present in the dental pulps are classified as mesenchymal, due to their ectomesenchymal embryonic origin, which gives them the ability to differentiate into a large number of specialized cells in the human body, such as bone, cartilage, muscle, neuronal cells, cardiac, pancreatic, among others. The youthfulness of these stem cells present in the pulps of deciduous teeth that will be lost and replaced by permanent dentition, approximately between 6 and 12 years of age, added to their expressive capacity for multiplication and plasticity in the laboratory, in addition to the high immunomodulatory potential, make this model stand out from other existing sources of mesenchymal stem cells (Fig 7).

**Figure 8:**
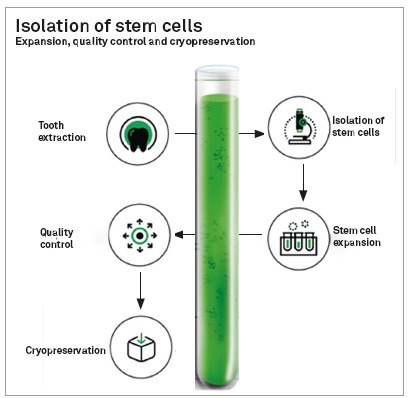
Infographic with sequence of events for cell cryopreservation.

The performance of the dental surgeon is essential as the homologator of this applied science, as responsible for monitoring the child and choosing the best tooth for performing the collection. The promotion and maintenance of oral health since childhood is a decisive step towards the viability of the pulp stem cell cryopreservation process. The dental unit chosen may be deciduous in the process of exfoliation, with 1/3 of the root volume remaining (the presence of the underlying permanent must be confirmed, with 2/3 of rhizogenesis completed) or a third molar in the process of rhizogenesis, so that the entire process of isolation, multiplication and validation of the stem cells to be cryopreserved is favored, in suitable conditions, for autologous use in advanced therapies, when and if necessary (Fig 8).

Stem cells from dental pulps express osteogenic markers and respond to many growth factors for osteo-odontogenic differentiation,^17^ ^-^ ^21^ and also multiply very easily and with quality in the laboratory.

There are many applications and potentials for the use of these stem cells, which go far beyond the boundaries of Dentistry itself. For Dentistry, specifically, the osteogenic potential and the ability to regenerate complex tissues - such as the pulp, periodontal ligament and the tooth itself - give the dentist new and powerful tools, which need to be presented to society in a responsible manner.

Cell Processing Centers fulfill the role of promoting the proper treatment and storage of these stem cells, following the internationally established protocols of good practice, so that they can be used when and if necessary, in autologous or allogeneic form, in line with current regulations established by the competent regulatory agents.

In Brazil, regulatory advances regarding the so-called advanced therapies, that make use of stem cells or factors obtained from these, have been happening more and more rapidly and consistently. The resolutions published by ANVISA (214/2018^22^ - Good Practices in Human Cells for Therapeutic Use and Clinical Research, and 260/2018^23^ - Rules for Conducting Clinical Trials with Advanced Investigational Therapy Product in Brazil), are an important demonstration of the environment favorable to the solid development of effectively innovative and transforming therapeutic protocols.

Cell Therapy brings the proposal to effectively and integrally regenerate tissues or organs morphologically, structurally and functionally damaged. Adult stem cells are presented as an important instrument, capable of leading and acting in this process in a decisive way. Countless basic and applied researches have been carried out all over the world, in order to prove the safety, efficacy, reproducibility, advantages and accessibility for the use of adult stem cells in Regenerative Medicine and Dentistry.^24^

## POSSIBLE CLINICAL APPLICATIONS OF MESENCHYMAL STEM CELLS IN DENTISTRY

### 1. Regeneration of pulp tissue

**Figure 9:**
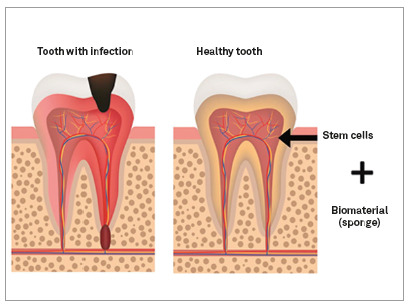
Illustration of pulp regeneration.

In Brazil, in 2015, a group from the Faculty of Dentistry of the University of São Paulo (USP) presented, in a preclinical study in rats, a complete regeneration of the pulp tissue after 28 days of the pulpectomy and inoculation of autologous stem cells at the site.^34^ In 2017, Nakashima et al.^25^ carried out the first clinical study with the purpose of evaluating the regenerative potential of pulp stem cells in pulp tissues affected by pulpitis, through autologous transplants. In this study, five patients diagnosed with irreversible pulpitis were submitted to endodontic treatment and then treated with autologous stem cells, with follow-up for 24 weeks. Pulp stem cells were associated with specific SDF1 factors (Factor 1 derived from stromal cells) in a collagen framework, since, according to the authors, the Regenerative Medicine triad - composed of progenitor cells, growth / migration factors and a framework - is essential for the “ideal” regenerative process to happen. After four weeks, they were able to verify a robust regenerative response, through electric pulp testing (EPT). At 24 weeks, through magnetic resonance imaging and computed tomography, they were able to observe the presence of regenerated pulp tissue and formation of functional dentin (Fig 9).

### 2. Expanding the limits of orthodontic movement

There are several factors that limit the extent of orthodontic movement,^26^ among which we can highlight the bone envelope, which is influenced by the pressure exerted by the adjacent soft tissues, neuromuscular forces and levels of periodontal insertion. Orthodontic movement is achieved by remodeling of the adjacent periodontal ligament in response to the load imposed from its constriction in the region influenced by the action of compressive orthodontic loads.^27^ Osteoclasts from hematopoietic stem cells^28^ are recruited to act directly in the bone remodeling process, accelerating the orthodontic movement.

The influence on orthodontic movement speed in relation to the expression of type-1 collagen by stem cells from the adjacent periodontal ligament was demonstrated, based on a study carried out in rats.^29^ The authors observed the suppression of local type-1 collagen, by stem cells of the periodontal ligament, during the application of orthodontic load, and an increase in local expression after the removal of this load.

**Figure 10:**
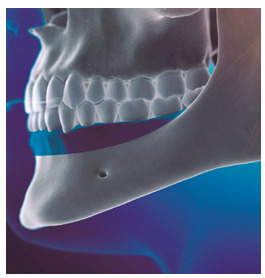
Expansion of orthodontic movement limits.

The use of stem cells can then represent an important window of opportunity for the bone remodeling process determined by the applied orthodontic forces to happen more quickly and predictably^27^ ^,^ ^29^ (Fig 10).

The tissue bioengineering strategy aimed at regenerating and/or increasing the bone ridge using mesenchymal stem cells from deciduous pulps associated with a biomaterial (framework) with osseoconductive, osseoinductive and bioactive properties is an excellent option to be considered in conditions ranging from the adequacy of the bone rim for orthodontics, to more extensive procedures aimed at orthopedics, such as dentofacial anomalies (cleft lip and palate), temporomandibular disorders, osteogenic distractions and maxillary expansions.

### 3. Periodontal regeneration

**Figure 11:**
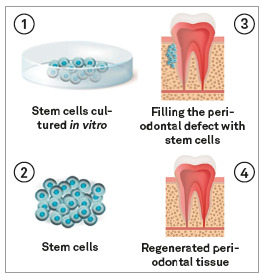
Mesenchymal stem cells for periodontal regeneration.

The periodontal phenotype must be considered with great attention in orthodontic planning, as cases of fenestration or gingival recession as a result of inappropriate orthodontic movement are not uncommon. However, once the framework is established, Guided Tissue Regeneration (GTR) techniques are a way to minimize the damage caused by the defect (Fig 11). Several authors have suggested that the path through GTR may have its results greatly improved if stem cells are associated with the process. Stem cells from the periodontal ligament have been especially considered in animal models^30^ ^,^ ^31^ and have been shown to be able to form specific periodontal structures when implanted in ectopic sites.^32^

### 4. External root resorption

**Figure 12:**
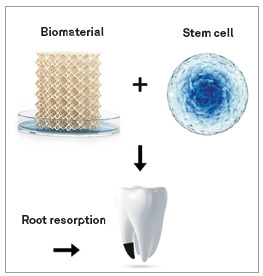
Mesenchymal stem cells undergoing root regeneration (external resorption).

This is a complication that often results from orthodontic treatments, and leads to loss of cementum and root dentin. The cementogenic potential of stem cells from the periodontal ligament and pericoronary follicle was investigated, considering that the cells that form the cementum are derived from these stem cells.^33^ The authors observed that stem cells from the periodontal ligament isolated by enzymatic digestion had special characteristics for cementogenesis. They were able to form, *in vivo*, a tissue similar to cell cementum, containing cells positive for osteocalcin. The other samples, on the other hand, formed materials similar to an acellular cementum. Considering that the defect in the root cementum must be regenerated from the deposition of new cell cementum, the use of stem cells from the periodontal ligament isolated by enzymatic digestion may constitute a path to be followed in therapeutic attempts for regeneration in cases of external resorption root (Fig 12).

## FINAL CONSIDERATIONS

The use of adult mesenchymal stem cells has been consolidated in the scientific and clinical communities, as an important tool for Regenerative Medicine and Dentistry. Therefore, it is necessary that applied research be strongly promoted, so that we have, in a short period of time, therapeutic possibilities increasingly efficient, safe, predictable and accessible to society without distinction. Clinicians face the challenge of disseminating information based on scientific evidence to the whole of society, giving the real dimension to this very important science. Dental surgeons are the only professionals qualified to choose and remove dental units for the purpose of cryopreservation of dental pulp stem cells. This exclusivity places Dentistry in a prominent position among the scientific community. Cell Processing Centers must continue to fulfill their important role of caring for and providing stem cells in conditions suitable for use in properly regulated research and advanced therapies. Finally, the competent regulatory entities must establish guidelines for good practices applied to laboratories, hospitals, clinics, therapy centers and professionals, while the Dentistry and Medicine Councils must coordinate, through the constitution of competent technical committees, the approval of the various possibilities of cell therapies that are described, proven and registered, in accordance with the international recommendations proposed by the main organized societies, such as the International Society for Cell and Gene Therapy (ISCT) and the International Society for Stem Cell Research (ISSCR).
